# An examination of the relationship between childhood abuse, anger and violent behavior among a sample of sex offenders

**DOI:** 10.1186/s40352-015-0025-3

**Published:** 2015-06-24

**Authors:** Stephanie R Ramirez, Elizabeth L Jeglic, Cynthia Calkins

**Affiliations:** Department: Forensic Psychology, Institute: John Jay College of Criminal Justice, 524 West 59th. Street, New York, NY 10019 USA

**Keywords:** Sex offender, Violence, Anger, Aggression, Childhood abuse

## Abstract

**Background:**

Increasing attention has focused on the emotional dysregulation that can result from adverse childhood experiences among those who commit sexually violent crimes. While studies confirm a relationship between child maltreatment and anger the research is limited and it is unclear how anger and child maltreatment effect the use of violence during the commission of the sex crime.

**Methods:**

This study examined the relationship between childhood maltreatment, anger and violent behavior by reviewing the records of 571 adult male offenders convicted of sexual assault or child molestation. The aims of the present study were to 1) examine differences in anger levels between those offenders who engaged in verbal or physical violence or used a weapon during the commission of their crime; 2) explore differences in anger levels for those sex offenders who experienced childhood abuse (physical abuse, sexual abuse, emotional abuse, and neglect) and those who were not; 3) examine whether there were differences in anger between rapists and child molesters and 4) assess whether anger either mediated or moderated the relationship between childhood abuse and the use of violence in the commission of the crime.

**Results:**

Overall we found that sex offenders who were rated as being angrier were more likely to have used violence in the commission of their crime and were more likely to be abused as children. Further, while these relationships held for both rapists and child molesters independently, rapists were found to be angrier than child molesters. Finally, anger neither mediated nor moderated the relationship between an offender’s adverse childhood and committing a violent sex crime.

**Conclusions:**

These results suggest that anger should be target in intervention and prevention programs with violent sex offenders.

## Background

The United States has one of the highest rates of reported sexual violence of any industrialized country (Stewart, 2002). An average of 237,868 people age 12 and older are raped and sexually assaulted each year (Federal Bureau of Investigation, 2012). Specifically, each year an average of 84,376 forcible rapes (penetration of the vagina or anus with any body part or object, or oral penetration by a sex organ of another person, without the consent of the victim) are reported to law enforcement (Federal Bureau of Investigation 2012) and it is estimated that this number is actually much higher as many sexual assaults go unreported (LeBreton et al. 2013). More than half (58 %) of sexual assault victims sustained a physical injury (e.g. cuts, bruises, internal injuries, broken bones, or gunshot wounds) during the attack (Federal Bureau of Investigation 2012). Further, about one in ten rapes or sexual assault victimizations involved the use of a weapon (Federal Bureau of Investigation 2012). Specifically, the offender possessed a firearm in 6 % of victimizations and a knife in 4 % of cases (Federal Bureau of Investigation 2012). Given the impact of sexual violence on society, it is imperative that clinicians understand the factors contributing to sexual violence in order to properly treat sex offenders who commit these violent crimes.

### Aggression in sex offenders

Criminal sexual behaviors by definition contain an element of aggression and coercion (Dalton et al. 1998). Apart from the variation in the type and level of aggression used in the commission of sexual offenses, the use of aggression may serve different purposes for different offenders (Smallbone and Milne, 2000). Aggression has traditionally been conceptualized as being either instrumental, an act of aggression that intends to hurt someone, but as a means to a goal other than causing pain or expressive, an act of aggression stemming from a feeling of anger and intended to cause pain or injury. It is theorized that these two types of aggression are precipitated by different subjective experiences and external circumstances in men who sexually offend (MSO) (Browne and Howells, 1996). Aggression then can be further dichotomized in physical and verbal aggression wherein physical aggression takes the form of pushing, hitting, kicking, or other means of physically fighting (Tremblay et al. 2005) and verbal aggression consists of words and insults, such as threats to injure or kill the victim (Crick et al. 2002).

Anger has been implicated as both a mediating (Betancourt and Blair, 1992; Dalton et al. 1998; Rebellon et al. 2012), and moderating (Pond et al. 2012; Sprague et al. 2011) factor for aggression and aggressive behavior. In addition anger has been cited as a motivator for criminal activities and reoffending (Walters, 1990); a personal attribute that puts an offender at a higher risk of reoffending (Andrews, 1996); and as an antecedent to interpersonal violence and serious violent crimes (Welsh & Gordon, 1991; Kroner et al. 1992). Offenders who engaged in aggression of any kind were less capable of controlling their behavior and more likely to experience anger than those who did not engage in aggression (Ramirez and Andreu, 2006; Smallbone and Milne, 2000). The likelihood of aggressive behavior following episodes of anger co-varied with the individual’s disposition to experience anger arousal (Tafrate and Kassinove, 2002).

Among MSO, those who were verbally aggressive and made death threats during the commission of their offenses were more disposed to perceive situations as anger provoking as compared to those MSO who did not overtly threaten their victim. Once angered, verbally aggressive MSO were more likely to express their anger outwardly and less likely to control their anger as compared to less verbally aggressive MSO (Smallbone and Milne, 2000). Another study found that anger levels were even higher for MSO convicted of sex crimes where excessive physical force was used as compared to offenders convicted of sex crimes who did not use excessive physical force (Rada et al. 1983).

In an effort to better understand the etiology of sex offending, clinicians developed a series of typologies in which anger featured prominently. Groth (1979) was one of the first researchers to describe the importance that anger has on offenders convicted of rape based upon the degree of aggression used, the underlying motivation of the offender, and the existence of other antisocial behaviors. He developed four typologies, later expanded upon by Berger (2000) (e.g. the power reassurance rapist, the power assertive rapist, the anger retaliation rapist, and the sadistic rapist). Of the four rapist typologies, the anger retaliation rapist constituted 40 % of the sample and according to Groth these types of rapists are driven to offend by the need for power, and feelings of anger and aggression (Groth, 1979). Their assaults are fueled by anger and characterized by excessive physical brutality, including degrading language and humiliating acts. Further, far more actual force is used in the commission of the offense than would be necessary if the intent was simply to overpower the victim and achieve sexual penetration (Groth 1979). Knight and Prentky (1990) developed classification system similar to Groth’s (1979), focusing on typologies of opportunistic, pervasively angry, vindictive and sexual rapists. According to Knight and Prentky’s (1990) classification system, pervasively angry rapists are motivated by anger, aggression, and hatred. In a follow-up study, Knight (1999) identified pervasive anger as one of four primary motivations among rapists, indicating that global and undifferentiated anger pervades all areas of the offender’s life. According to this model, pervasively angry sex offenders express anger and aggression in their sexual assaults, which results in physical injury to the victim. These rapists may use violence even if the victim does not resist. Although these rapes highlight the role of anger in sex offending, the research supporting the link between anger and violent behavior is largely based upon clinical descriptions with limited empirical support (Loza and Loza-Fanous, 1999).

While the role of anger in sexual offenses has been described for rapists, little is known about the role of anger among those who are convicted of sexual crimes against children. One study found that anger expression varied depending on the type of sex crime conviction, but that the role of anger remained a key feature underlying MSO as a group (Lee et al. 2001). Specifically MSO who offended against children (MSO-CM) suppressed or turned anger inward, rapists (MSO-R) turned anger outward, and MSO who committed crimes against both children and adults both suppressed and acted out their anger (Dalton et al. 1998; Hudson and Ward, 1997; Kalichman, 1991; Lee et al. 2001; Rada et al. 1983). Available studies (Mann and Hollin, 2001; Milner and Webster, 2005; Ward, 2000) on schemata in sexual offenders have found different key themes in MSO-CM and MSO-R that can be related to anger expression. Schemas of hostility to women, sexual entitlement, and a need for control have been found in MSO-R whereas MSO-CM presented a greater sense of worthlessness. Carvalho and Nobre (2013) found that compared to MSO-CM and non-offenders, MSO-R presented higher levels of negative affect. It has been suggested that MSO overall may use sex to reduce negative emotions and improve positive emotions (Ward and Beech, 2006). More recently, studies have shown a relationship between early maladaptive schemas (EMSs) and sexual aggression among convicted MSO (Carvalho and Nobre, 2013; Chakhssi et al. 2013). Indeed, EMSs are vulnerability factors for later psychological/personality problems being strongly related to emotional, interpersonal, and behavioral difficulties (Young et al. 2003). Taken together these studies suggest that anger is a salient feature of psychopathology for individuals convicted of sex crimes but the precursors triggering anger need to be explored.

### History of child maltreatment

The childhood histories of offenders convicted of sex crimes are characterized by high rates of physical abuse, sexual abuse, and/or dysfunctional family relations (Ainsworth, 1989; Beech and Mitchell, 2005; Dhawan and Marshall, 1996; Gannon et al. 2008; Haapasalo and Kankkonen, 1997; Marshall and Barbaree, 1989). More specifically, childhood histories of sexually violent offenders are characterized by neglect, violence, and disruption within the home (Bard et al. 1987; Craissati and McClurg, 1996).

Adverse childhood experiences are associated with differential sexual offending behaviors (Haapasalo and Kankkonen, 1997; Knight and Prentky, 1990; Lee et al. 2001; Simons et al. 2008; Simons et al. 2002). Sexual abuse during childhood is related to the severity of sexual aggression, whereas physical abuse and neglect are associated with the severity of nonsexual aggression (Knight and Prentky, 1990). It is evident that not all victims of sexual abuse become perpetrators, and not all perpetrators have experienced childhood abuse which suggests that the experience of sexual abuse appears to be neither a necessary nor sufficient condition for committing a sex crime (Salter et al. 2003). Sexual abuse alone does not cause violent sexual behavior, but a pattern of experiences consisting of physical abuse and emotional rejection alongside sexual abuse may increase the risk that male victims of sexual abuse become abusers themselves.

MSO often report a childhood history of physical abuse, suggesting that victims of physical abuse may learn to behave violently in their home environment thereby translating that violence into their interpersonal relationships as children and later as adults (Simons et al. 2008). Developing a predisposition for violence is a potential consequence of having been a victim of physical abuse (Mass et al. 2008). Researchers have found physically abused boys were more likely to be charged with sexual offenses during adolescence and arrested for violent sex crimes, such as rape later in life than boys who were not physically abused (Kobayashi et al. 1995; Widom and Ames, 1994). Compared to MSO-CM and nonsexual offenders, MSO-R reported more frequent experiences of physical and emotional abuse, and paternal violence (Connoly and Woollons, 2008; Smallbone and Dadds, 1998). Findings from a meta-analysis conducted by Jespersen et al. (2009) showed that a sexual victimization history was significantly more prevalent among MSO-CM than MSO-R. Widom et al. (2006) found a direct path from childhood victimization to sexual violence in a sample of men whose cases were drawn from criminal court records. Directly, child abuse and neglect were associated with higher levels of violent arrests. Indirectly, child abuse and neglect were significantly predictive of violent arrests through early aggression. In the overall model, child abuse, both physical and sexual, and neglect were both directly and indirectly predictive of arrests for sexually violent crimes.

Emotional abuse and psychological maltreatment have also been suggested as possible developmental precursors for sexual violence (Simons et al. 2008). The negative effects of a child’s perception development when experiencing maltreatment is dependent upon the degree of perceived damage that the child experiences (Simons et al. 2008). Children who frequently experienced emotional abuse exhibited higher rates of physical aggression and interpersonal problems later in life (Teicher et al. 2006).

Children who are victims of one form of abuse are more likely to also experience other forms of abuse (Edwards et al. 2003; Mullen et al. 1996). Specifically it appears that the greater the number of forms of abuse experienced, the more severe the subsequent pathology (Dube et al. 2001; Edwards et al. 2003). This then increases the propensity to experience and act upon feelings of anger. Adverse outcomes worsen for a child who has repeatedly experienced multiple types of child maltreatment; however, even maltreatment of a limited duration appears to have a lasting impact for negative behaviors later in life. No single factor can account for the development of violent sexual behavior (English et al. 2002; Mass et al. 2008; Maxfield and Widom, 1996; Smith and Thornberry 1995; Zingraff et al. 1993), however, victims of childhood abuse appear to be at risk for deleterious physical and psychological consequences increasing one’s chances for crime and violence later in life and thus potential mechanisms through which child maltreatment results in sexual violence need further exploration.

### Current study

The relationship between anger and violent behavior among offenders convicted of sex crimes has been controversial. While some researchers reported the existence of a link between the two (Andrews, 1996; Howells, 1989; Howells, 2004; Kroner et al. 1992; Novaco, 1994; Walters, 1990; Welsh and Gordon, 1991; Zamble and Quinsey, 1997), others have disputed it (Loza and Loza-Fanous, 1999; Tice and Baumeister, 1993;). There are indications that the emotion of anger is an important influence on offending for some MSO, but the mechanisms that fuel this relationship remain unclear. Increasing attention has been paid to the emotional dysregulation that can result from experiencing childhood abuse (Eckhardt et al. 2008; Gratz et al. 2009) since anger is a common reaction to traumatic exposure, (Andrews et al. 2000; Brewin et al. 2000; Connor et al. 2003) and high levels of anger have been reported in adulthood among individuals who were physically and/or sexually abused as children (Feeny et al. 2000; Ruch et al. 1991). Further, high levels of anger reported among offenders who were physically and/or sexually abused as children suggest that anger may act as a contributing factor in the commission of violent sex crimes. Further there appear to be differences between the experience and expression of anger between rapists and child molesters. Thus the goal of the current study was first to examine the role of anger among MSO. Specifically we sought to examine whether anger was related to verbal aggression, physical aggression and weapon use during the commission of the crime. Next we examined the relationship between childhood abuse and anger. Then in an effort to better understand the developmental mechanisms behind violent sexual behavior we explored whether anger was a possible mediator or moderator in the relationship between childhood abuse and the commission of a violent sex crime. In addition, given the differences between MSO-R and MSO-CM we examined whether the aforementioned relationships differed by offender type. Based upon the previous literature it was hypothesized that 1) those MSO whose crimes involved verbal and physical aggression as well as weapon use would be rated higher on measures of anger; 2) those who report a history of childhood abuse would receive higher anger scores; and 3) anger would mediate or moderate the relationship between an offender’s history of childhood abuse and the commission of a violent sex crime. Finally we hypothesized the MSO-R and MSO-CM would express anger and aggression differently such that anger would be more related to the crimes of MSO-R than those of MSO-CM.

## Method

### Participants and procedure

The data for this study was gathered from a larger study of offenders convicted of sex crimes (*n* = 3168) who were housed in either a prison-based sex offender treatment facility or the general population of a state prison (Mercado et al. 2011). Records of offenders who were released between 1996 and 2007 were reviewed and coded by trained MA level research assistants. Inter-rater reliability was calculated for 10 % of the files and there was substantial agreement between researchers’ rating of dichotomous variables (*k* = .621-.788) and excellent level of agreement between researchers on pervasive anger scores (ICC = .924). All procedures were approved by the university and department of corrections institutional review boards.

The current sample was comprised of only adult male offenders convicted of rape, or child molestation and who received a score ranging from 0–5 (*M* = .93, *SD* = 1.30) on a pervasive anger measure scored by trained clinicians (See Table 1). The majority of the offenders, 64.1 % (n = 366) were housed in a prison-based sex offender treatment facility while 35.7 % (n = 204) of the offenders were in the general population of the state prison. Only a subset of the entire sample were administered the pervasive anger measure and thus they comprised the final sample of 571 MSO.

**Table 1:** Demographics of the sex offender population

	All (n = 571)	Rapists (n = 124)	Child molesters (n = 438)
*Anger*	227 (39.8 %)	76 (61.3 %)	146 (33.3 %)
*Violence*	122 (21.4 %)	74 (59.7 %)	44 (10.1 %)
*Verbal*	171 (30 %)	79 (63.7 %)	87 (19.9 %)
*Weapon*	87 (15.3 %)	64 (51.6 %)	20 (4.6 %)
*Hx of abuse*	224 (41.6 %)	44 (36.7 %)	178 (43.4 %)

Offenders ranged in age from 18 to 74 (*M* = 35, *SD* = 11.01) at the time they entered prison. Offenders came from diverse racial and ethnic backgrounds including 234 Whites (41 %), 240 African Americans (42 %), 88 Latinos (15.4 %), 3 Asian Pacific Islanders (.5 %), and 4 were of Other (.7 %) ethnicity. The ethnicities of two offenders were unknown (.4 %). The sample consisted of individuals with an index offense conviction of rape (*n* =124), child molestation (*n* = 438), or both rape and child molestation (*n* = 9). Of this sample, 463 offenders victimized females, 86 offenders victimized males, and 18 offenders victimized both males and females. There were eight files missing victim gender. When looking at offender’s prior histories and convictions, 284 offenders had a history of prior charges and/or convictions, while 278 offenders did not have a prior history, while nine offenders’ prior histories were unknown.

### Measures

#### Pervasive anger

For the purpose of this study, pervasive anger was defined as the total score on a measure assessing expressive anger in 5 domains for offenders convicted of rape and child molestation: (1) Angry person who easily loses temper or anger directed at multiple targets in multiple situations; (2) Consistent pattern of verbal aggression against both males & females; (3) Assaults against males and frequent (2 or more occasions) physical fights with males; (4) Offender reports preoccupation with aggressive fantasies that include thoughts of beating, killing, etc.; and (5) Offender has been cruel to animals, which includes having beaten, tortured or killed them. This measure was designed at the treatment center and was scored by the treating clinician. Each of the aforementioned items were coded dichotomously (yes/no) to represent the presence or absence of this trait based upon the treating clinician’s judgment. An offender’s total pervasive anger score represented the number of anger items endorsed by the clinician.

#### Violence during the commission of the crime

Three different types of violence curing the commission of the crime were assessed: verbal aggression, physical aggression and weapon usage. All information was retrieved and coded from the offenders’ prison records. Verbal abuse was dichotomously (yes/no) coded if the offender threatened the victim during the commission of the offense. Physical violence was also dichotomously (yes/no) coded if the index crime involved slapping, punching, or hitting, while weapon usage was dichotomously (yes/no) coded if the offender utilized a weapon during the commission of a crime.

#### Adverse childhood history

Childhood abuse was categorized into four types consisting of physical abuse, sexual abuse, psychological abuse (including verbal, mental, and emotional abuse) and neglect. Information was retrieved from the offender’s prison files.

## Results

Descriptive statistics for the dependent measures are presented in Tables 1 and 2. The mean on the Pervasive Anger measure was .93, *SD* = 1.30 (range 0–5). Further, 39.1 % (*n* = 223) of the sample reported a history of childhood abuse. Within the sample, 30 % (n = 171) offenders used verbal violence to threaten their victim(s), 21.4 % (n = 122) offenders used physical violence, 15.3 % (n = 87) offenders used a weapon, and 380 offenders did not use any act of violence to threaten or harm their victim(s).

**Table 2:** Means and standard deviation for descriptive variables

	All		Rapists		Child molesters	
	M	SD	M	SD	M	SD
Pervasive Anger	.93	1.30	1.60	1.50	.73	1.16
Childhood Abuse	1.11	1.42	2.02	1.56	.86	1.27
No Childhood Abuse	.79	1.10	1.28	1.38	.64	1.07
Verbal Aggression	1.54	1.46	1.80	1.49	1.32	1.38
No Verbal Aggression	.66	1.14	1.24	1.48	.58	1.06
Physical Aggression	1.80	1.52	1.97	1.52	1.45	1.42
No Physical Aggression	.69	1.13	1.04	1.31	.64	1.10
Weapon Use	1.77	1.44	1.77	1.40	1.95	1.64
No Weapon Use	.77	1.22	1.42	1.60	.67	1.11

To test whether there were group differences in perceived anger scores between those who used verbal aggression, physical force or who used a weapon during the commission of the crime a series of t-tests were conducted. Significant differences in anger were found between those who used verbal aggression *t* (262.36) = −7.02, *p* < .01, two tailed, *d* = .67; physical aggression *t* (158.80) = −7.56, *p* < .01, two tailed*, d* = .83; and weapon usage *t* (109.24) = −6.05, *p* < .01, two tailed*, d* = .75 such that those who used verbal aggression, physical aggression or who used weapons were rated higher on perceived anger.

To test whether there were group differences in perceived anger scores between MSO-R and MSO-CM a separate t-test was conducted. We found significant differences in pervasive anger scores between offenders *t* (166.86) = 5.95, *p* < .01, two tailed, *d* = .65; such that MSO-R were rated significantly higher on pervasive anger than MSO-CM. Further, we found that MSO-R were more likely to engage in verbal aggression, χ^2^ = (1, *n* = 561) = 86.86 *p* = .00, *phi* = −.40; physical aggression, χ^2^ = (1, *n* = 561) = 140.15 *p* = .00, *phi* = −.50; and weapon usage, χ^2^ = (1, *n* = 561) = 164.18 *p* = .00, *phi* = −.55 than MSO-CM.

Next we tested whether offender type (MSO-R and MSO-CM) impacted differences in pervasive anger scores by type of violence used during the commission of the crime. To do this we conducted a separate series of t-tests for MSO-R and MSO-CM. For the MSO-R group we found significant differences in pervasive anger scores between those who used verbal aggression *t* (92.08) = −2.00, *p* = .05, two tailed*, d* = .38; and physical aggression *t* (114.82) = −3.55, *p* < .01, two tailed*, d* = .66; such that those who used verbal aggression and physical aggression during the commission were rated significantly higher on pervasive anger. There was no significant difference in pervasive anger scores for MSO-R who used a weapon *t* (122) = −1.30, *p* = .198, two tailed, *d =* .23*.*

Among the MSO-CM group, significant differences were found in pervasive scores between those who used verbal aggression *t* (112.40) = −4.68, *p* < .01, two tailed*, d* = .60; physical aggression; *t* (48.96) = −3.66, *p* < .01, two tailed*, d* = .64 and weapon use *t* (19.84) = −3.46, *p < .*01, two tailed, *d* = .91; where those who used verbal and physical abuse and used weapons were rated higher on the pervasive anger measure.

### Childhood abuse and anger

In order to explore the relationship between childhood abuse and anger an independent sample t-test was conducted to compare the Pervasive Anger scores for the population of MSO who have a history of childhood abuse and those who do not (yes/no). There was a significant difference in scores for offenders with a history of abuse (*M* = 1.11, *SD* = 1.42) and without a history of abuse (*M* = .79, *SD* = 1.10; *t* (421.25) = −2.70, *p* = .01, two tailed*, d* = .25).

The relationship between childhood abuse and anger (as measured by the Pervasive Anger Scale) for the population of offenders convicted of sex crimes as a whole was further investigated using a Pearson product–moment correlation coefficient where childhood abuse was measured as a continuous variable, with those experiencing multiple types receiving higher scores. There was a small, positive correlation between the two variables, *r* = .12, *n* = 537, *p* < .01, with high levels of pervasive anger associated with childhood abuse.

The relationship between childhood abuse and anger for MSO-R and MSO-CM independently was further investigated. There was a significant difference in anger scores for MSO-R with a history of abuse (*M* = 2.02, *SD* = 1.56) and offenders convicted of rape without a history of abuse (*M* = 1.28, *SD* = 1.38; *t* (118) = −2.72, *p* = .01, two tailed*, d* = .50). Further, using abuse as a continuous variable there was a small, positive correlation between the two variables, *r* = .22, *n* =120, *p* < .05, with high levels of pervasive anger associated with childhood abuse for offenders convicted of rape. Among MSO-CM there was no significant difference in anger scores for offenders convicted of child molestation with a history of abuse (*M* = .86, *SD* = 1.27) and without a history of abuse (*M* = .64, *SD* = 1.07; *t* (344.51) = −1.88, *p* = .06, two tailed*, d* = .19) and further there was no significant correlation between pervasive anger and childhood abuse assessed continuously, *r* = .09, *n* = 410, *p* > .05.

### Anger as a mediator

To test the hypothesis that pervasive anger mediates the relationship between an individual’s childhood history of abuse for offenders convicted of rape and/or child molestation and committing a violent sex crime, a mediation model was tested. In Step 1 of the mediation model Sexual Violence was regressed on childhood abuse and was not found to be significant *b* = −0.007, *p* = .91. The relationship between childhood abuse and the mediator variable of anger, was significant *b* = 0.032, *t*(571) = 2.78, *p* = .006; as with the mediator variable of anger, and the outcome variable of violence (*b* = 0.386, *p* < .01). Therefore pervasive anger does not act as a mediator between a sex offender’s history of abuse and committing a violent sex crime, but maltreatment produces anger and anger produces violence in sex offenders.

### Anger as a moderator

Utilizing Baron and Kenny’s (1986) method a moderation model was tested to determine if pervasive anger moderated the relationship between a history of childhood abuse and committing a violent sex crime. A hierarchal multiple regression analysis was conducted. In step one, childhood abuse did not account for a significant amount of variance in committing a violent sex crime, *R* ^*2*^ = .00, *F* (1, 535) = .01, *p* = .91. In step two, when pervasive anger was added into the equation, both anger and childhood abuse accounted for a significant amount of variance in committing a violent sex crime, *R* ^*2*^ = .11, *F* (2, 534) = 33.01, *p* < .01. In step three, the interaction term between childhood abuse and pervasive anger was added to the regression model and did not account for a significant proportion of the variance in committing a violent sex crime, *ΔR* ^*2*^ = .113, *F* (1, 533) = 22.619, *p* = .207. Therefore, pervasive anger was not found to moderate the relationship between a sex offender’s history of abuse and committing a violent sex crime, but an offender’s total pervasive anger alone does predict violence.

## Discussion

An emerging issue in the field of sex offender theory and treatment is whether emotional and other affective states function as antecedents for offenses (Howells et al. 2004). This study assessed the relationship among several variables related to emotional under-control of anger in offenders convicted of sex crimes. Specifically we examined whether there were differences in anger levels of MSO who used verbal and physical aggression or weapons during the commission of the crime. In addition, we studied the relationship between childhood abuse and anger. We also examined whether these relationships differed between MSO-R and MSO-CM. Finally we investigated whether anger functioned as a mediator or moderator in the relationship between childhood abuse and violence committed during the crime. Overall we found that MSO who used violence in their crimes were rated as being angrier than those MSO who did not. Further we found that those MSO who had a history of childhood abuse were also assessed to be angrier. While both of these relationships appeared to hold true for both MSO-R and MSO-CM, MSO-R were rated as angrier than MSO-CM. Finally, we found that anger does not mediate nor moderate the relationship between a MSO’s history of childhood abuse and committing a sexually violent crime.

As expected MSO who used violence during the commission of the crime were significantly angrier than MSO who did not use violence. These findings support the notion that the emotion of anger has a significant impact on offender’s crime scene behavior such that it may serve as motivational precursor for verbal aggression and physical aggression among MSO (Rada et al. 1983; Smallbone and Milne, 2000).

Consistent with previously established findings (Andrews et al. 2000; Brewin et al. 2000; Connor et al. 2003; Paivio and Laurent, 2001; Ruch et al. 1991; Springer et al. 2007) the MSO in the present study who have a history of childhood abuse had a significantly higher anger score than MSO without a history of abuse. Even though anger is an emotion that is commonly experienced by individuals who are victims of abuse as well as those with no abuse history, the way it is expressed tends to differ between victims and non-victims (Maneta et al. 2012). Various mechanisms have been proposed to explain the link between childhood abuse and risk for future violence. In recent research, increasing attention has been paid to the emotional dysregulation that can result from childhood abuse experiences (Gratz et al. 2009). While many sex offender treatment programs include an anger management component, researchers have argued that it is not necessary and should be removed. Our research suggests that for those offenders who have experienced abuse, or who have engaged in violent offenses, anger management may indeed be warranted. Further analyses explored the impact childhood abuse has on anger in offenders convicted of rape and/or child molestation.

As expected, offenders in this study who experienced multiple types of abuse had a significantly higher anger score than offenders who experienced only one type of abuse. Clinicians have noted marked distinctions in the dynamics of hostility, dominance, and dependency between individuals convicted of child molestation and individuals convicted of rape. Weak empirical evidence was reported in previous research on anger and aggression in offenders convicted of sex crimes resulting in inconsistencies throughout the literature (Smallbone and Milne, 2000). Significant progress has been made in identifying predictors among violent behavior in offenders convicted of rape and/or child molestation. The present study highlights the importance of considering multiple types of abuse when examining the effect of anger on violent sexual behavior in offenders convicted of sex crimes. Research has documented that the combination of multiple types of maltreatment were associated with negative adult health outcomes one of which was increased anger (Springer et al. 2007). This study supports these findings and suggests that this relationship holds true for both MSO-R and MSO-CM.

Consistent with previous research (Hall and Hirschman, 1991; Lee et al. 2001; Marshall and Barbaree, 1990) MSO-R and MSO-CM do differ significantly in their expression and experience of anger as well as the amount of violence used in the sexual assault. The sex offender literature (Knight and Prentky, 1990; Pithers, 1989; Yates et al. 1984, Zamble and Quinsey, 1997) suggests that offenders who commit sex crimes against adults report experiencing feelings of anger prior to the commission of rape whereas offenders who commit sex crimes against children may use denial and repression as a primary means of coping with and avoiding anger. The majority of offenders in this sample were convicted of child molestation. In the absence of decisive evidence regarding the link between anger and sexually violent behavior, our results support previous research which suggests that anger management strategies may be warranted for both MSO-R and MSO-CM. Unfortunately, denial and repression, and other forms of emotional over-control, which are more common in offenders who commit sex crimes against children were not assessed on the Pervasive Anger Measure.

Anger did not serve as a mediator or moderator between an offender’s history of childhood abuse and committing a violent sex crime in this sample. Previous studies examined anger as a mediating and moderating variable for violent offenders in terms of violent crime behavior (Betancourt and Blair, 1992; Dalton et al. 1998; Rebellon et al. 2012) but very few studies specifically examined a population of offenders convicted of sex crimes. This study was one of the first to look at anger among offenders convicted of sex crimes with and without a history of childhood abuse as a mediating and moderating variable when committing a violent sex crime. However the sample of offenders convicted of sex crimes was disproportionately comprised of individuals convicted of child molestation, who as previously noted are less likely to express anger. In accordance with the sex offender literature (Smallbone and Milne, 2000), offenders convicted of child molestation used more verbal aggression than excessive physical force when angered, which could have negatively affected the number of violent sex crimes in this sample. Regardless, of the sample chosen, anger had a significant effect when examined with verbal aggression, physical aggression, weapon usage, and committing a violent sex crime. Anger was only associated with childhood abuse for offenders convicted of rape but not child molestation. One possible explanation for this finding could be that the majority of offenders experienced instrumental aggression, rather than expressive aggression, which was not measured on the Pervasive Anger Scale. An offender convicted of rape and/or child molestation does not require a high level of trait anger for state anger to be present or a necessary condition for the offense to occur (Howells et al. 2004). Because trait anger is associated more closely with expressive, than with instrumental aggression, offenders with a higher than average pervasive anger score may benefit better in anger management interventions, which are designed to reduce expressive or hostile aggression (Browne and Howells, 1996; McDougall et al. 1987). This may be due to the Pervasive Anger Scale only measuring expressive forms of anger. Anger as a mediating or moderating precursor for sexual violence is an important factor for clinicians when developing treatment plans because one must acknowledge both instrumental and expressive forms of anger when assessing the offender before developing an effective treatment plan.

### Limitations and future research

There are several limitations to the current study. Since these analyses were based upon archival review, the data available in the files were often not complete. As with any archival study of this nature, the data was not collected for research purposes, but rather was data maintained in prison files for sentencing, treatment, and management purposes. Thus the validity and accuracy of the results are dependent upon what information was entered and coded from the offenders file. Further in many instances the history of childhood abuse was not officially documented but rather reported by the offender. While there is a concern for potential fabrications, research suggests that the overall bias is in the opposite direction. Individuals are more likely to minimize or deny their adverse childhood experiences (Brewin et al. 1993). Out of the original sample (*N* = 3,168) a total of 571 offenders convicted of sexual assault, child molestation, or both received a score from the treating clinician on the pervasive anger measure limiting the results to a much smaller sample of MSO. Offenders were not matched based on any criteria nor was a control sample used. Because offenders were not matched based on any criteria there is an increased chance that factors other than childhood abuse (e.g. gang membership, psychiatric history, alcohol/drug problem) influenced the offender’s anger score. In addition, the measure of pervasive anger was not validated. This was a measure comprised of 5 clinician rated items pertaining to the general construct of anger. This has both advantages and drawbacks. Specific concerns regarding self-report measures are their vulnerability to lying, manipulation, and self-presentation biases (Kroner et al. 2007) and thus clinician reports may be a more valid observation based upon historical data and behavioural observation, but that since the scale is not validated it is unclear how closely this scale measures the construct. Clinicians’ rating offender’s pervasive anger may have been influenced by the offender’s crime scene behavior when rating anger. Further although this study attempted to provide a large-scale analysis of the mediating/moderating effect of anger between childhood abuse and violent sexual behavior, it should be noted that these are results from offenders convicted of sex crimes in one state, and the generalizability of the sample to other states is unclear.

The assumption that individuals convicted of sex crimes are likely to have problems with the experience and expression of anger is reflected in the widespread use of anger management strategies in sexual offender treatment programs (Marshall and Eccles, 1991; Sapp and Vaughn 1991). However, the associated assumption that sexual offending involves expressive forms of aggression has received very little empirical attention (Smallbone and Milne, 2000). Although limited, our research adds to the growing literature on the role of affect in sex offending. Despite the fact that the majority of the sample did not use violence, our research supports the assumption that anger is associated with aggression in the commission of violent sex acts. Due to the distinct differences in anger expression between MSO-R and MSO-CM our research sets the groundwork for future studies to measure both instrumental and expressive forms of anger in order to capture both emotional overcontrol and underregulation of negative affect in offenders convicted of sex crimes.

Few studies confirm anger as a mediating or moderating factor for violent behavior among offenders convicted of rape and/or child molestation leaving much room for further exploration. Given the current lack of epidemiological, prevention, and intervention research examining expressive forms of aggression in sexual offending (Smallbone and Milne, 2000), it is important for future research to further examine these factors. Future research is needed to control for differences among offenders when examining the effect anger has on violent sexual behavior. For the future, a focus on duration of abuse in childhood should be examined in relation to an offender’s anger score. It has been suggested that poor emotional attachments, both in the past and present, are the primary mechanisms by which victims become offenders of sex crimes (Browne and Herbert, 1997; Falshaw et al. 1996). When looking at anger in offenders convicted of rape and/or child molestation, future research should also account for mental illness and the effects anger may have on mentally ill offenders convicted of sex crimes. It is apparent that the link between anger and violence, already recognized as complex, could be influenced and confounded by many variables. Further empirical and theoretical attention given to the roles of trait anger and aggression in sexual offending may lead to further clarification of the link between anger and sexual violence as well as successful developments in the treatment and prevention of violent sexual offending.
